# Determination of μ-, δ- and κ-opioid receptors in forebrain cortex of rats exposed to morphine for 10 days: Comparison with animals after 20 days of morphine withdrawal

**DOI:** 10.1371/journal.pone.0186797

**Published:** 2017-10-20

**Authors:** Hana Ujcikova, Martina Hlouskova, Kristina Cechova, Katerina Stolarova, Lenka Roubalova, Petr Svoboda

**Affiliations:** 1 Department of Biomathematics, Institute of Physiology of the Czech Academy of Sciences, Prague, Czech Republic; 2 Department of Biochemistry, Faculty of Science, Charles University in Prague, Prague, Czech Republic; Temple University, UNITED STATES

## Abstract

**Background:**

Chronic exposure of mammalian organism to morphine results in adaption to persistent high opioid tone through homeostatic adjustments. Our previous results indicated that in the frontal brain cortex (FBC) of rats exposed to morphine for 10 days, such a compensatory adjustment was detected as large up-regulation of adenylylcyclases I (8-fold) and II (2.5–fold). The other isoforms of AC (III-IX) were unchanged. Importantly, the increase of ACI and ACII was reversible as it disappeared after 20 days of morphine withdrawal. Changes of down-stream signaling molecules such as G proteins and adenylylcyclases should respond to and be preceded by primary changes proceeding at receptor level. Therefore in our present work, we addressed the problem of reversibility of the long-term morphine effects on μ-, δ- and κ-OR protein levels in FBC.

**Methods:**

Rats were exposed to increasing doses of morphine (10–40 mg/kg) for 10 days and sacrificed either 24 h (group +M10) or 20 days (group +M10/−M20) after the last dose of morphine in parallel with control animals (groups −M10 and −M10/−M20). Post-nuclear supernatant (PNS) fraction was prepared from forebrain cortex, resolved by 1D-SDS-PAGE under non-dissociated (−DTT) and dissociated (+DTT) conditions, and analyzed for the content of μ-, δ- and κ-OR by immunoblotting with C- and N-terminus oriented antibodies.

**Results:**

Significant down-regulation of δ-OR form exhibiting M_w_ ≈ 60 kDa was detected in PNS prepared from both (+M10) and (+M10/−M20) rats. However, the total immunoblot signals of μ-, δ- and κ-OR, respectively, were unchanged. Plasma membrane marker Na, K-ATPase, actin and GAPDH were unaffected by morphine in both types of PNS. Membrane-domain marker caveolin-1 and cholesterol level increased in (+M10) rats and this increase was reversed back to control level in (+M10/−M20) rats.

**Conclusions:**

In FBC, prolonged exposure of rats to morphine results in minor (δ-OR) or no change (μ- and κ-OR) of opioid receptor content. The reversible increases of caveolin-1 and cholesterol levels suggest participation of membrane domains in compensatory responses during opioid withdrawal.

**General significance:**

Analysis of reversibility of morphine effect on mammalian brain.

## Introduction

The receptors for opioid drugs, μ-OR, δ-OR, κ-OR and NOP-OR, belong to the rhodopsin family of G-protein-coupled receptors (GPCRs). All of these receptors inhibit adenylyl cyclase (AC) activity through activation of the G_i_/G_o_ class of trimeric G proteins. G_i_/G_o_ proteins are present in the brain in large quantities and inhibit adenylyl cyclase activity or regulate ionic channels in a pertussis toxin-dependent manner [1–3]. Morphine and related μ-OR agonists represent the most effective drugs for relief of acute as well as chronic pain [4, 5].

Results from our previous study [6] indicated, in accordance with data from Sim et al. [7], Sim-Selley et al. [8], and Maher et al. [9], a significant desensitization of DAMGO- and DADLE-stimulated G-protein responses in plasma membranes (PM) isolated from forebrain cortex of rats exposed to increasing doses of morphine (10–50 mg/kg) for 10 days (group +M10). In these membranes, ACI and ACII were up-regulated 8-fold and 2.5-fold, respectively, when compared with membranes prepared from control animals [10]. The increase in ACI and ACII was specific as the amount of ACIII-IX, Na, K-ATPase and trimeric G-protein α and β subunits remained the same as baseline. Importantly, the elevation in ACI and ACII was not detected in membranes isolated from animals that received morphine for 10 days and were subsequently nurtured for 20 days in the absence of the drug (group +M10/–M20). Thus, the marked increase in ACI and ACII faded away 20 days after the last dose of morphine.

Up-regulation of ACI and ACII manifested in the rat forebrain cortex after 10 days of morphine exposure may be regarded as a compensatory “over-shoot” mechanism that has been noticed previously by others [2, 11–15]. The disappearance of this “over-shoot” after 20 days of morphine withdrawal indicates the ability of an organism to return to physiological norms after cessation of an (in this case, opioid) drug supply [10]. This idea was recently supported by proteomic analysis that indicated the total number of proteins altered by morphine in rat forebrain cortex by more than 2-fold was dramatically reduced after 20 days of morphine withdrawal [16].

Our subsequent work using resolution of PM proteins by two-dimensional electrophoresis (2D-ELFO) and identification by liquid chromatography-mass spectrometry (LC-MS/MS) demonstrated that trimeric G-protein β subunits (Gβ) were reduced 2-fold in PM samples prepared from morphine-treated rats. However, the morphine-altered pool of Gβ subunits, when resolved in 2D gels and identified with immunoblot analysis, represented just a small fraction (at pI ≈ 5.6) of the total signal of Gβ subunits which was distributed over the wide range of pI (≈ 4.5–6.8). The decrease in the total signal of Gβ subunits (1.2-fold) was not significant. This result implied the idea that just a small fraction of signaling proteins residing in the plasma membrane may respond to morphine exposure whilst the majority of PM population of this protein is not affected [17].

Secondary changes in downstream signaling molecules of OR-signaling cascades, such as G proteins or adenylylcyclases, should respond to and be preceded by primary changes at the opioid receptor level. Therefore, in our present work, we addressed the problem of reversibility of long-term morphine effects on μ-, δ-, and κ-OR protein levels in rat forebrain cortex. To obtain comparable results with our previous data [10,16,17], rats exposed to morphine for 10 days (group +M10) were evaluated against those exposed to morphine for 10 days and subsequently nurtured for 20 days in the absence of the drug (group +M10/–M20). 1D-SDS-PAGE in 10% gels or 4–12% NuPAGE gradient gels was employed for resolution of the post-nuclear supernatant fraction (PNS) and opioid receptors determined by immunoblotting with C-terminus and N-terminus oriented antibodies. The specificity of morphine effect on OR level was tested in parallel samples of PNS by determination of prototypical PM marker Na, K-ATPase, actin and GAPDH as negative standards. Caveolin-1 was measured as markers of membraine domains/lipid rafts.

## Materials and methods

### Chemicals

Acrylamide and bis-acrylamide were from SERVA (Heidelberg, Germany). Immobiline DryStrips, Pharmalyte buffer (broad pH range 3–10) and Immobiline DryStrip cover fluid were purchased from GE Healthcare (Piscataway Township, NJ). Complete protease inhibitor cocktail was from Roche Diagnostic (Mannheim, Germany), [^3^H]-ouabain (30 mCi/mmol, NET211001MC) from Perkin Elmer and *N-*glycosidase F (11 365 169 001, Roche) was from Sigma-Aldrich. All others chemicals were of highest purity available and purchased from Sigma-Aldrich (St. Louis, USA).

### Antibodies

The μ-, δ- and κ-opioid receptors were identified by rabbit polyclonal antibodies purchased from Santa Cruz Inc. (Dallas, USA): MOR-1 (C-20, sc-7488-R, C-terminus, 1:1000 dilution), MOR-1 (H-80, sc-15310, N-terminus, 1:1000 dilution), DOR-1 (H-60, sc-9111, N-terminus, 1:1000 dilution) and KOR-1 (H-70, sc-9112, N-terminus, 1:1000 dilution). Antibodies oriented against α subunit of Na, K-ATPase (H-300, sc-28800, 1:1000 dilution), caveolin-2 (H-96, sc-7942, 1:1000 dilution), actin (I-19, sc-1616, 1:1000 dilution) and GAPDH (FL-335, sc-25778, 1:2000 dilution) were also from Santa Cruz Inc. Caveolin-1-oriented antibody was from Transduction Laboratories (C13630-050, 1:5000 dilution). The Human endothelial cell lysate was used as a positive control for Caveolin-1 (Transduction Laboratories). Donkey anti-rabbit IgG-HRP (NA934V, 1:40000 dilution) was from GE Healthcare UK and goat anti-rabbit IgG-HRP (sc-2004, 1:10000 dilution) was from Santa Cruz Inc.

### Morphine treatment of experimental animals

Young adult male Wistar rats (220–250 g) were exposed to morphine by intra-muscular application according to the following protocol: 10 mg/kg (days 1 and 2), 15 mg/kg (days 3 and 4), 20 mg/kg (days 5 and 6), 30 mg/kg (days 7 and 8) and 40 mg/kg (days 9 and 10). The morphine-treated rats were sacrificed 24 h (group +M10) or 20 days (group +M10/ −M20) after the last dose of morphine. Control animals were injected with sterile PBS and sacrificed in parallel with morphine-treated rats, i.e. 24 h (group −M10) or 20 days (group −M10/ −M20) after the last dose of morphine. Each experimental group was represented by 6–7 animals. Food (Altromin standard diet, Germany) and drinking water was provided *ad libitum*. Rats were housed in groups of 2–3 in standard boxex enriched with Lignocel (Hygienic Animal Bedding) and maintained on a 12 h ligh/dark cycle. The temperature was 22±2°C with a humidity 55±5%.

Animals were killed by decapitation under ether narcosis, the frontal brain was rapidly removed and washed intensively from the remaining blood with ice-cold saline (145 mM NaCl). The frontal brain cortex was separated on the pre-cooled plate, snap frozen in liquid nitrogen and stored at −80°C until use. The experiments were approved by Animal Care and Use Committee of the Institute of Physiology of the Czech Academy of Sciences to be in agreement with Animal Protection Law of the Czech Republic as well as the European Communities Council Directive (86/609/EEC).

### Preparation of post-nuclear supernatant (PNS) and plasma membrane (PM) fractions from rat forebrain cortex

The tissue pieces were minced with razor blade on pre-cooled plate, diluted in STEM medium (250 mM sucrose, 20 mM Tris-HCl, 1 mM EDTA, 3 mM MgCl_2_, pH 7.6) containing 1 mM fresh PMSF plus protease inhibitor cocktail, homogenized mildly in loosely-fitting Teflon-glass homogenizer for 5 min (2 g w. w. per 10 ml) and centrifuged for 5 min at 3500 rpm (1200 × *g*). PNS fraction, represented by 1200 × *g* supernatant, was snap frozen in liquid nitrogen and stored at– 80°C.

For preparation of PM fraction, PNS was applied on top of 30 ml of 27.4% Percol in Beckman Ti70 tubes and centrifuged for 60 min at 30000 rpm (65000 × g). Centrifugation resulted in the separation of two layers [18]. The upper layer represented PM fraction; the lower layer contained mitochondria. The upper layer was removed, diluted 1:3 in STEM medium and centrifuged in Beckman Ti70 rotor for 90 min at 50000 rpm (175000 × g). Membrane sediment was removed from the compact, gel-like sediment of Percoll and re-homogenized in small volume of 50 mM Tris-HCl, 1 mM EDTA, pH 7.7 (TE buffer).

### 1D-SDS-PAGE and immunoblotting

The aliquots of PNS were mixed 1:1 with 2-fold concentrated Laemmli buffer (SLB) with (+DTT) or without (–DTT) 1 mM dithiothreitol and heated for 3 min at 100°C. Standard (10% w/v acrylamide/0.26% w/v bis-acrylamide) SDS electrophoresis was carried out as described before [17,19]. Molecular mass determinations were based on pre-stained molecular mass markers (Sigma, SDS 7B).

After SDS-PAGE, proteins were transferred to nitrocellulose (Protran BA 83, GE Healthcare) and blocked for 1 h at room temperature in 5% (w/v) low-fat milk in TBS-Tween buffer (10 mM Tris-HCl, pH 8.0, 150 mM NaCl, 0.1% (v/v) Tween 20). Primary antibodies were added in TBS-Tween containing 1% (w/v) low-fat milk and incubated for at least 2 h, then removed and the membrane was washed extensively (3 × 10 min) in TBS-Tween. Secondary antibodies (donkey or goat anti-rabbit IgG conjugated with horseradish peroxidase) were diluted in TBS-Tween containing 1% (w/v) low-fat milk, applied for 1 h and after three 10 min washes, the blots were developed by ECL technique using Super Signal West Dura (Pierce) as substrate. The developed blots were scanned with an imaging densitometer Epson Perfection 4990 Photo and quantified by Aida Image Analyzer v. 3.28 (Ray test).

The same samples of PNS were also analyzed by the NuPAGE system (Invitrogen). Aliquots of PNS were solubilized in NuPAGE LDS Sample Buffer (4x) with (─DTT) or without (+DTT) by addition of NuPAGE Sample Reducing Agent (10x) according to manufacturer’s instructions. Samples were heated at 40°C for 5 minutes, loaded at 10–20 μg/well and resolved in NuPAGE 4–12% gels using 3-(N-morpholino) propane sulfonic acid (MOPS), sodium dodecyl sulfate (SDS) running buffer with NuPAGE Antioxidant prior to blotting on nitrocellulose membranes. Western blotting was carried out as described above.

### Sample preparation for isoelectric focusing

Samples of PNS containing 2 mg protein were precipitated with ice-cold aceton overnight at– 20°C. After centrifugation at 9000 × *g* for 20 min at 4°C, the supernatant was removed and the pellet was extracted with ice-cold 6% TCA for 1.5 h on ice. After centrifugation at 9000 × *g* for 10 min at 4°C, the supernatant was discarded and the pellet washed with 600 μl of ice-cold 96% ethanol for 1 h at room temperature. The mixture was centrifuged at 9000 × *g* for 10 min at 4°C and the remaining pellet was solubilized with 250 μl IEF sample buffer containing 7 M urea, 2 M thiourea, 4% CHAPS, 1% DTT, 1% ampholines pH 3–10 and 0.01% bromphenol blue for 3 h at room temperature. After a brief centrifugation (9000 × *g*, 1 min), samples were transferred into a groove of the Immobiline DryStrip Reswelling Tray (GE Healthcare).

### Two-dimensional electrophoresis (2D-ELFO)

Immobiline DryStrips (linear pH gradient 3–11 NL, 13 cm) were placed into the Immobiline DryStrip Reswelling Tray containing protein samples and rehydrated overnight. Isoelectric focusing was performed using the Multiphor II system (GE Healthcare) at 14°C in the following manner: 150 V for 5 h, 500 V for 1 h, 3500 V for 12 h and 500 V for 3 h. The focused strips were stored at– 20°C or immediately used.

The strips were rinsed thoroughly with ultrapure water, dried quickly on filter paper and equilibrated in 5 ml of equilibration buffer (50 mM Tris-HCl pH 6.8, 6 M urea, 0.1 mM EDTA, 2% SDS, 30% glycerol and 0.01% bromophenol blue) containing 1% DTT for 10 min in order to reduce disulphide bridges and other oxidized groups. Subsequently, the strips were alkylated in equilibration buffer containing 2.5% iodoacetamide for 10 min. Molecular weight markers were loaded onto a piece of filter paper and placed close to the alkaline side of the strip. The strip and molecular marker were covered with 0.5% agarose. Gels were run vertically at a constant current of 20 mA for 20 min and then at 90 mA for 4 h till the bromophenol blue dye reached the end of the gel. The apparatus was cooled to 15°C using the Hoefer SE 600 unit (GE Healthcare). After SDS-PAGE, proteins were transferred to nitrocellulose (1300 mA, 2.5 h) by using TE62 Standard Transfer Tank (Hoefer), blocked in 5% (w/v) low-fat milk /TBS-Tween, washed 3x in TBS-Tween, incubated with horseradish peroxidase-conjugated Ab and developed by ECL. The developed blots were scanned with an imaging densitometer Epson Perfection 4990 Photo and quantified by Aida Image Analyzer v. 3.28 (Ray test).

### Treatment with *N-*glycosidase F

The stock solution of *N*-glycosidase F in 50 mM sodium phosphate, 12 mM EDTA and 50% glycerol (v/v), pH 7.2 was used according to the following protocol: PNS samples containing 200 μg protein were transferred into a 1.5 ml tube and diluted with ultrapure water to reach the final volume of 45 μl. Protein denaturation by SDS was performed by addition of 2.5 μl of 2% SDS and incubation for 5 min at 100°C. Samples were put on ice and treated with 2.5 μl of 15% Triton X-100 in order to avoid inactivation of this enzyme by SDS. 3 μl of *N*-glycosidase F were added and samples were incubated for 3 h at 37°C. Subsequently, SDS-PAGE and immunoblotting were performed as described above.

### Na, K-ATPase

All samples of PNS were tested for the content of the prototypical plasma membrane marker, Na, K-ATPase (EC 3.6.1.3). This was made by resolution by SDS-PAGE (+DTT) in 10% gel and immunoblotting with antibody oriented against α-subunit of this enzyme (sc-28800, Santa Cruz) as described by Dlouha et al. [19].

The content of Na, K-ATPase was also determined as the number of specific [^3^H]ouabain binding sites at saturating concentration of this inhibitor [20]. PNS samples were diluted to the same protein concentration in control (─M10, ─M10/─M20) and morphine-treated (+M10, +M10/─M20) samples and incubated with 18 nM [^3^H]ouabain for 90 min at 30ºC in total volume of 0.2 ml of 5 mM NaHPO_4_, 5 mM MgCl_2_, 50 mM Tris-HCl, pH 7.6. The binding reaction was terminated by dilution with 5 ml of ice-cold buffer and filtration through Whatman GF/B filters. The filters were washed twice and radioactivity remaining on the filters was determined by liquid scintillation using Rotiszcint Eco Plus cocktail. Non-specific binding was determined in the presence of 10 μM unlabelled ouabain.

### [^3^H]naloxon and [^3^H]diprenorphine binding

With aim to determine the total density of μ-, δ- and κ-OR in PNS and PM fractions, specific [^3^H]naloxon (non-selective antagonist) and [^3^H]diprenorphine (non-selective agonist) binding was measured similarly as described before by Richie and Noble [21] and Ko et al. [22]. The PNS (360 μg protein) or PM (100 μg protein per tube) fraction was incubated in final volume of 100 μl of binding mix containing 50 mM Tris–HCl, 1 mM EDTA, pH 7.7 and the saturating, 5 nM concentration of [^3^H]naloxon or [^3^H]diprenorphine. Specific radioligand binding (B_sp_) was obtained as the difference between binding in the absence (B_t_) and presence (B_nsp_) of nonradioactive 100 μM naloxone. After incubation for 90 min at 30°C, samples were diluted with 3 ml of ice-cold TE buffer, immediately filtered and washed twice with 3 ml of TE buffer. Whatman GF/B filters mounted in the Brandel cell harvester were used for separation of bound and free radioactivity. Radioactivity remaining on the filters was determined by liquid scintillation.

### Cholesterol

Cholesterol level in PNS was determined by Amplex Red cholesterol kit (Molecular Probes, Eugene, OR) according to the manufacturer’s protocol.

### Protein determination

Lowry method was used for determination of protein concentration in all samples of PNS using bovine serum albumin (Sigma, Fraction V) as a standard. Data were calculated by fitting the calibration curve as a quadratic equation.

### Statistical analysis

The significance of the difference between control and morphine-treated samples of PNS was analyzed by Student´s *t*-test and GraphPad*Prism4*. Results represent the average ± SEM.

## Results

### Determination of μ-OR level in frontal brain cortex of rats exposed to morphine for 10 days; comparison with animals sacrificed 20 days since morphine withdrawal

**Fig 1:**
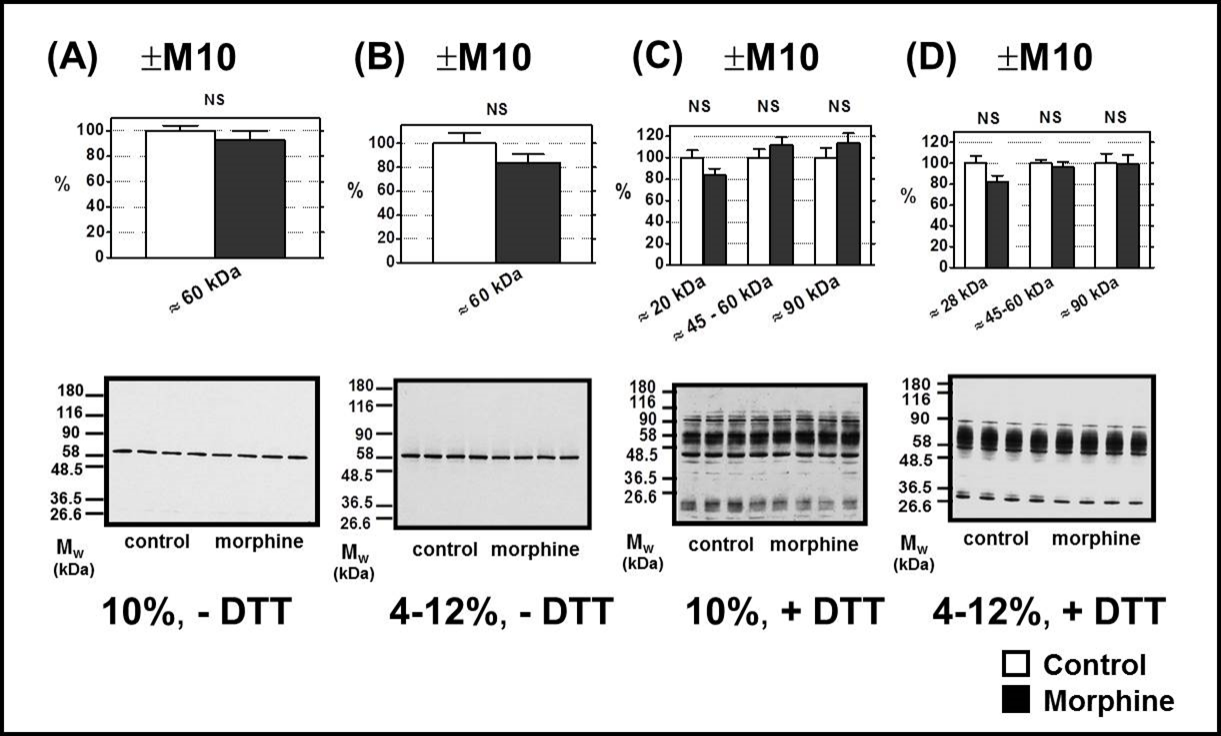
Determination of μ-OR in PNS prepared from FBC of experimental groups (+M10) and (─M10). PNS fractions (20 μg protein per lane) prepared from morphine-treated (+M10) and control (— M10) rats were resolved under non-dissociated (— DTT) or dissociated (+DTT) conditions by 1D-SDS-PAGE in 10% w/v acrylamide/0.26% w/v bis-acrylamide (**A, C)** or in 4–12% NuPAGE gels (**B, D**); μ-OR was recognized by C-terminus-oriented Ab C-20 (sc-7488-R). When resolved under non-dissociated conditions (**A** and **B**), Ab C-20 distinguished the single protein with M_w_ ≈ 60 kDa. The average intensity of this immunoblot signal was not significantly different when compared in control (— M10) and morphine-treated (+M10) PNS samples (NS, p>0.05). Resolution under dissociated conditions (**C** and **D**) revealed the presence of multiple protein bands exhibiting a wide range of M_w_ ≈ 20–90 kDa. These immunoblot signals were also unchanged by morphine (NS, p>0.05). The results (upper columns) represent the average signal of five immunoblots, each performed with four control + four morphine-treated samples of PNS ± SEM. 100% on y-axis represents the average intensity of a given immunoblot signal determined in PNS prepared from control, (─M10) rats. The significance of difference between the control and morphine-treated PNS samples was analyzed by Student´s *t*-test using GraphPad*Prizm4*. In the lower panels, typical immunoblots are presented.

The single, strong immunoblot signal of μ-OR with M_w_ of ≈ 60 kDa was detected by C-terminus-oriented Ab C-20 (sc-7488-R) from Santa Cruz in PNS samples prepared from rat FBC which were resolved by 1D-SDS-PAGE in 10% w/v acrylamide/0.26% w/v bis-acrylamide (**Fig 1A)** as well as 4–12% NuPAGE gradient gels (**Fig 1B)** in the absence of DTT (─DTT). This result was in agreement with data provided by the supplier–the single μ-OR protein signal with M_w_ ≈ 50 kDa was detected by these antibodies in mouse brain tissue extract (compare with **S1 Fig**). Thus, the specificity of Ab C-20 was sufficient for recognition and quantitative determination of μ-OR level in rat FBC by immunoblotting. The average intensity of this μ-OR signal was not significantly different when compared in (+M10) and (─M10) samples of PNS (**Fig 1A** and **1B**).

Resolution of PNS proteins under dissociated conditions (+DTT) revealed the presence of multiple μ-OR protein bands exhibiting a wide range of M_w_: from 20 to 90 kDa (**Fig 1C** and **1D**). As in resolution under non-dissociated conditions, the signal of these proteins was not significantly different when compared in control and morphine-treated samples of PNS. The immunoblot signals were scanned and quantified individually in areas corresponding to M_w_ ≈ 20 kDa, 45–60 kDa and ≈ 90 kDa and the average intensity of these signals (collected from the five immunoblots, each performed with four control + four morphine-treated samples), was not significantly altered by morphine (p > 0.05, NS). The same result was observed with the assessment of PNS samples prepared from rats sacrificed 20 days since the last dose of morphine (group +M10/─M20) when compared with corresponding control animals (group ─M10/─M20). The lack of morphine effect on μ-OR level in rat brain cortex was also found by De Vries et al. [23] when using [^3^H]DAMGO binding assay for determination of μ-OR (see discussion for more details).

The μ-OR signals detected at low M_w_ (<30 kDa) were also noticed by other groups [24–26]. It is reasonable to assume that these low M_w_ bands represent a degraded forms of μ-OR arising from native μ-OR (M_w_ ≈ 44 kDa; data sheet of supplier) by proteolytic cleavage. Surprisingly, this hydrolytic cleavage takes place in spite of the fact that fresh PMSF together with a protease inhibitor cocktail were present before the onset of homogenization of the brain tissue.

The protein bands with artificially low M_w_ (<30 kDa) were also seen when μ-OR was discerned by N-terminus-oriented Ab H-80 and these signals were not influenced by morphine, either. In 45–90 kDa area of the resolving 10% or 4–12% gradient gels, the Ab H-80 yielded just the faint signals which were impossible to use for reproducible quantification. Thus, the affinity of Ab H-80 was too low for satisfactory determination of this type of OR in rat forebrain cortex (data not shown).

### δ-OR and κ-OR in FBC of rats exposed to morphine for 10 days; comparison with animals sacrificed 20 days since morphine withdrawal

**Fig 2:**
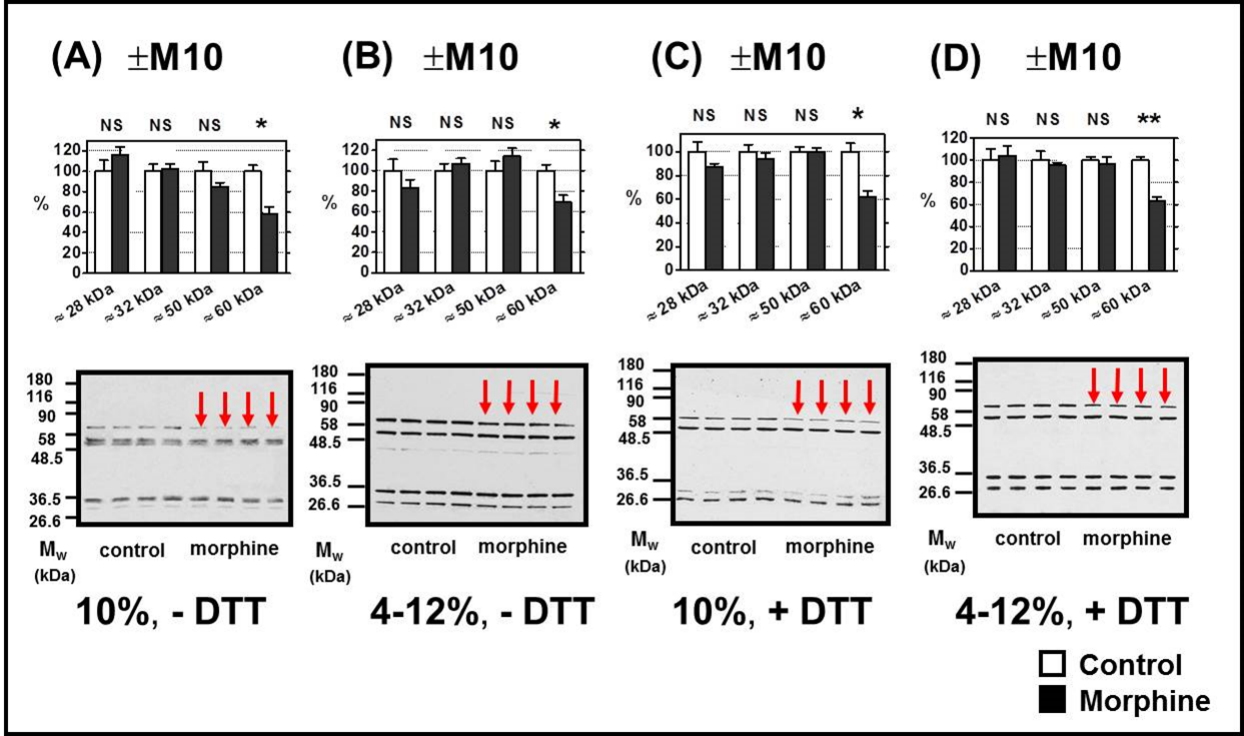
Determination of δ-OR in PNS prepared from FBC of experimental groups (+M10) and (─M10). PNS fractions (20 μg protein per lane) prepared from morphine-treated (+M10) and control (— M10) rats were resolved under non-dissociated (— DTT) or dissociated (+DTT) conditions by 1D-SDS-PAGE in 10% w/v acrylamide/0.26% w/v bis-acrylamide (**A, C)** or in 4–12% NuPAGE gels (**B, D**) and δ-OR was recognized by N-terminus-oriented Ab H-60 (sc-9111). Significant down-regulation (↓) of δ-OR isoform with M_w_ ≈ 60 kDa was detected (*, p<0.05). Intensities of immunoblot signals of other forms of δ-OR detected at ≈ 28 kDa, ≈ 32 kDa and ≈ 50 kDa were unchanged by morphine (NS, p>0.05). The results (upper columns) represent the average signal of five immunoblots each performed with four control + four morphine-treated samples of PNS ± SEM. 100% on y-axis represents the average intensity of a given immunoblot signal determined in PNS prepared from control, (─M10) rats. The significance of difference between the control and morphine-treated PNS samples was analyzed by Student´s *t*-test using GraphPad*Prizm4*. In the lower panels, typical immunoblots are shown.

The N-terminus oriented antibodies Ab H-60 (sc-9111) were fully appropriate for detection of δ-OR. The specifity of Ab H-60 was high as the single immunoblot signal of these antibodies was observed in HEK293 cells stably expressing δ-OR (**S2 Fig**). The intensity and clear-cut resolution of immunoblot signals produced by Ab H-60 in rat FBC was sufficient for quantitative analysis of δ-OR content in PNS samples prepared from this tissue (**Fig 2**). The four immunoblot signals with M_w_ ≈ 28 kDa, ≈ 32 kDa, ≈ 50 kDa and ≈ 60 kDa were recognized.

The intensity of δ-OR immunoblot signals detected at ≈ 28 kDa, ≈ 32 kDa and ≈ 50 kDa was not significantly different when compared in (+M10) and (─M10) samples of PNS, p > 0.05. The same result was obtained for the total signal of all δ-OR proteins (≈ 28 kDa, ≈ 32 kDa, ≈ 50kDa and ≈ 60 kDa)—the total signal was decreased to 87 ± 4% of control value (100%), but this decrease was not significant, p > 0.05.

The only exception was represented by δ-OR isoform with M_w_ ≈ 60 kDa. The actual M_w_ of this protein in different gels was slightly above M_w_ of pyruvate kinase from rabbit skeletal muscle (58 kDa), which was used as a M_w_ marker. The ≈ 60 kDa isoform of δ-OR was significantly decreased in morphine-treated samples (+M10) of PNS when compared with controls (— M10), p < 0.05. Importantly, the decrease of this δ-OR signal was noticed under dissociated (+DTT) as well as non-dissociated (─DTT) conditions when resolved PNS proteins by 1D-ELFO in both 10% and 4–12% gradient gels (**Fig 2**). Statistical analysis was based on data collected from five immunoblots, each containing four control and four morphine-treated samples of PNS. Exactly the same amount of protein (20 μg) was applied per lane.

The significant decrease of ≈ 60 kDa isoform of δ-OR was also noticed in PNS prepared from FBC of rats exposed to morphine for 10 days and subsequently nurtured for 20 days without morphine (group +M10/─M20).

**Fig 3:**
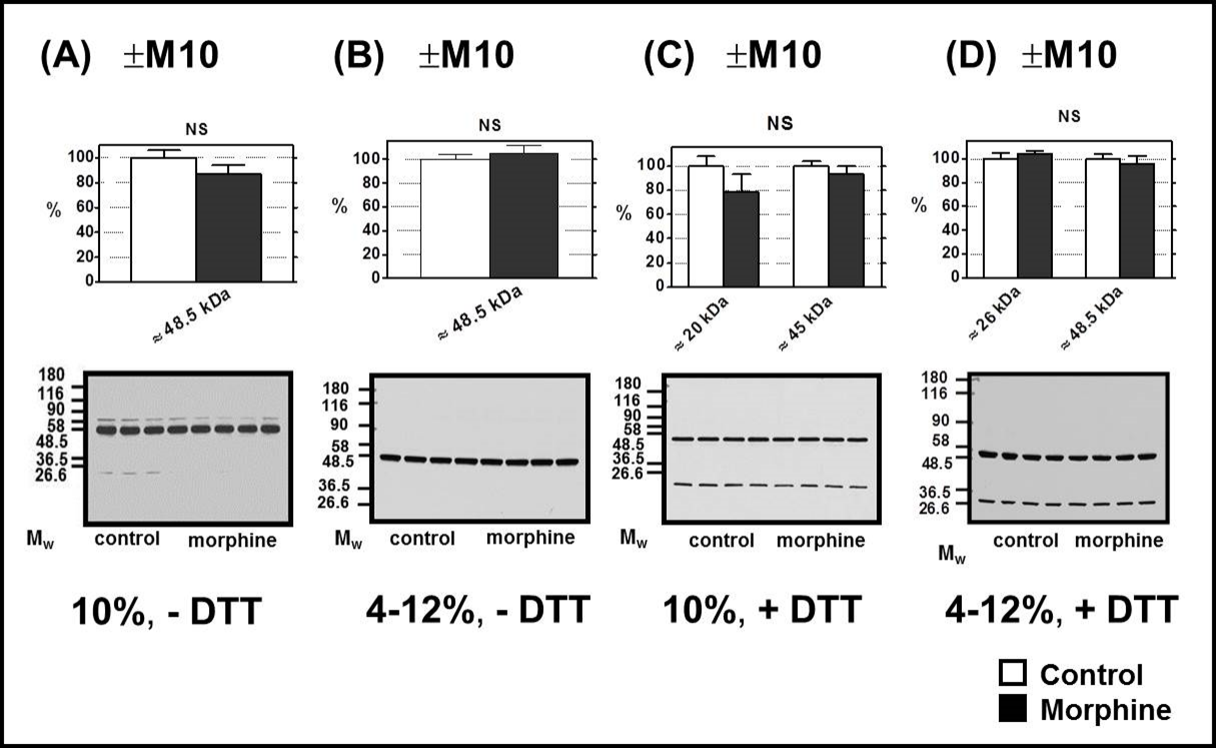
Determination of κ-OR in PNS prepared from FBC of experimental groups (+M10) and (─M10). PNS fractions (20 μg protein per lane) prepared from morphine-treated (+M10) and control (— M10) rats were resolved under non-dissociated (— DTT) or dissociated (+DTT) conditions by 1D-SDS-PAGE in 10% w/v acrylamide/0.26% w/v bis-acrylamide (**A, C)** or in 4–12% NuPAGE gels (**B, D**); κ-OR was recognized by Ab H-70 (sc-9112). The results (upper columns) represent the average signal of five immunoblot experiments performed with four control + four morphine-treated samples of PNS ± SEM. 100% on y-axis represents the average intensity of a given immunoblot signal determined in PNS prepared from control, (─M10) rats. The significance of difference between the control and morphine-treated PNS samples was analyzed by Student´s *t*-test using GraphPad*Prizm4*. In the lower panels, typical immunoblots are presented (NS, p>0.05).

**Fig 4:**
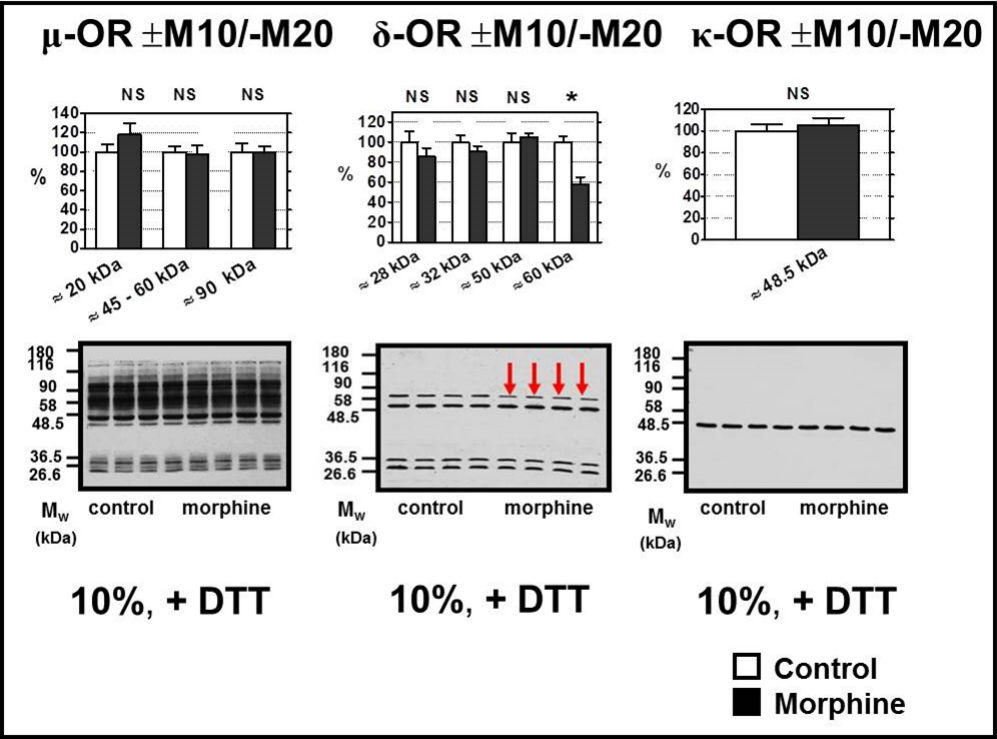
Determination of μ-, δ- and κ-OR in PNS prepared from experimental groups (+M10/─M20) and (─M10/─M20). PNS fractions prepared from (+M10/─M20) and (─M10/─M20) groups of rats were resolved under dissociated (+DTT) conditions by 1D-SDS-PAGE in 10% w/v acrylamide/0.26% w/v bis-acrylamide gels and the opioid receptors were recognized by antibodies from Santa Cruz: μ-OR (C-20, sc-7488-R, C-terminus), δ-OR (H-60, sc-9111, N-terminus), and κ-OR (H-70, sc-9112, N-terminus). As before, 20 μg of PNS protein was applied per each lane. The results (upper columns) represent the average signal of four immnoblots ± SEM. 100% on y-axis represents the average intensity of a given immunoblot signal determined in PNS prepared from control, (─M10) rats. The significance of difference between the (+M10/─M20) and (─M10/─M20) PNS samples was analyzed by Student´s *t*-test using GraphPad*Prizm4*. In the lower panels, typical immunoblots are shown (NS, p>0.05; *, p<0.05).

The intensity of the single strong signal of κ-OR recognized by Ab H-70 (compare with **S3 Fig**) at M_w_ ≈ 50 kDa in FBC was not altered by morphine in (+M10) samples of PNS (**Fig 3**); the same result was obtained by analysis of immunoblots performed with PNS prepared from (±M10/─M20) groups of rats (**Fig 4,** right panels).

### [^3^H]naloxon and [^3^H]diprenorphine binding

**Fig 5:**
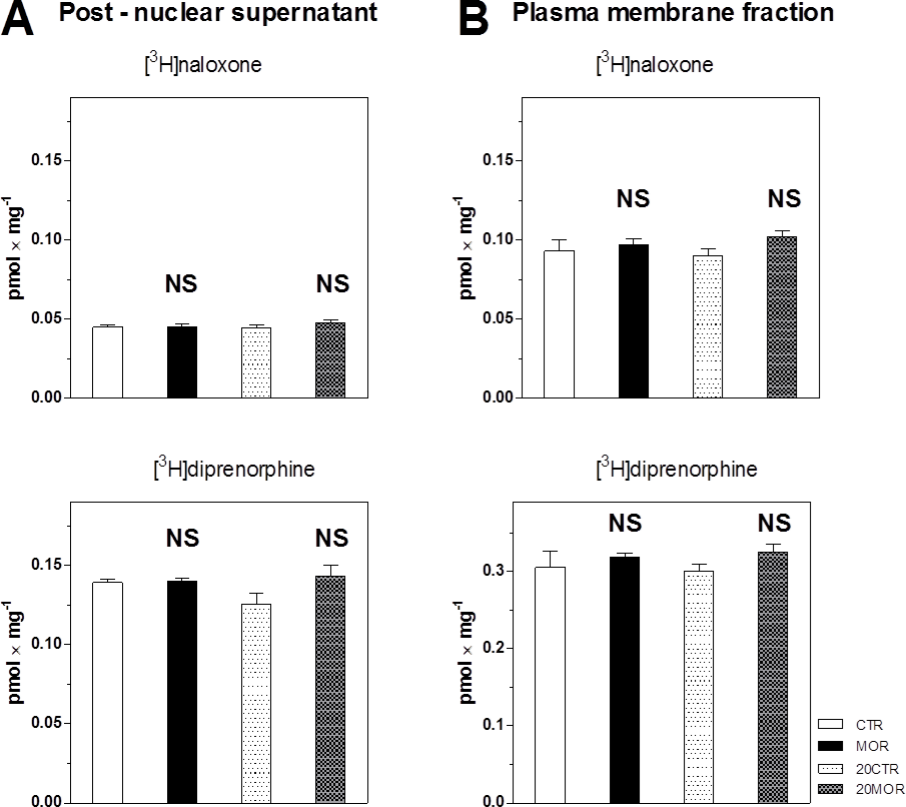
Density of [^3^H]naloxon and [^3^H]diprenorphine binding sites in PNS and PM fractions. **A.** [^3^H]naloxon (upper) and [^3^H]diprenorphine (lower panels) binding was measured in PNS fractions preparared from CTR (─M10), MOR (+M10), 20CTR (─M10/─M20) and 20MOR (+M10/─M10) groups of rats as described in Methods. Results represent the average of three binding assay each performed in pentaplicates. Significance of difference between the control and morphine-treated samples was analyzed by Student´s *t*-test using GraphPad*Prizm4*. **B.** [^3^H]naloxon (upper) and [^3^H]diprenorphine (lower panel) binding was measured in PM fractions prepared from the same groups of experimental animals as described in legend to **Fig 1A**. NS, p>0.05.

The immunoblot analysis of μ-, δ- and κ-OR content in FBC of control and morphine-treated rats was extended by radioligand binding assays using the non-selective OR ligands [^3^H]naloxon (antagonist) and [^3^H]diprenorphine (agonist). Determination of number of the specific binding sites at saturating concentration of these radioligands gives the immnoblot-independent information about total density of OR in a given sample of PNS. Results presented in **Fig 5A** indicated that the number of μ+δ+κ-OR binding sites for both these radioligands was not altered by morphine in (±M10) as well as (±M10/─M20) samples of PNS. The same results were obtained when the [^3^H]naloxon and [^3^H]diprenorphine binding assays were carried-out in PM fractions isolated from FBC of the same groups of animals (**Fig 5B).**

### μ-OR, δ-OR and κ-OR in PNS samples treated with *N*-glycosidase F

**Fig 6:**
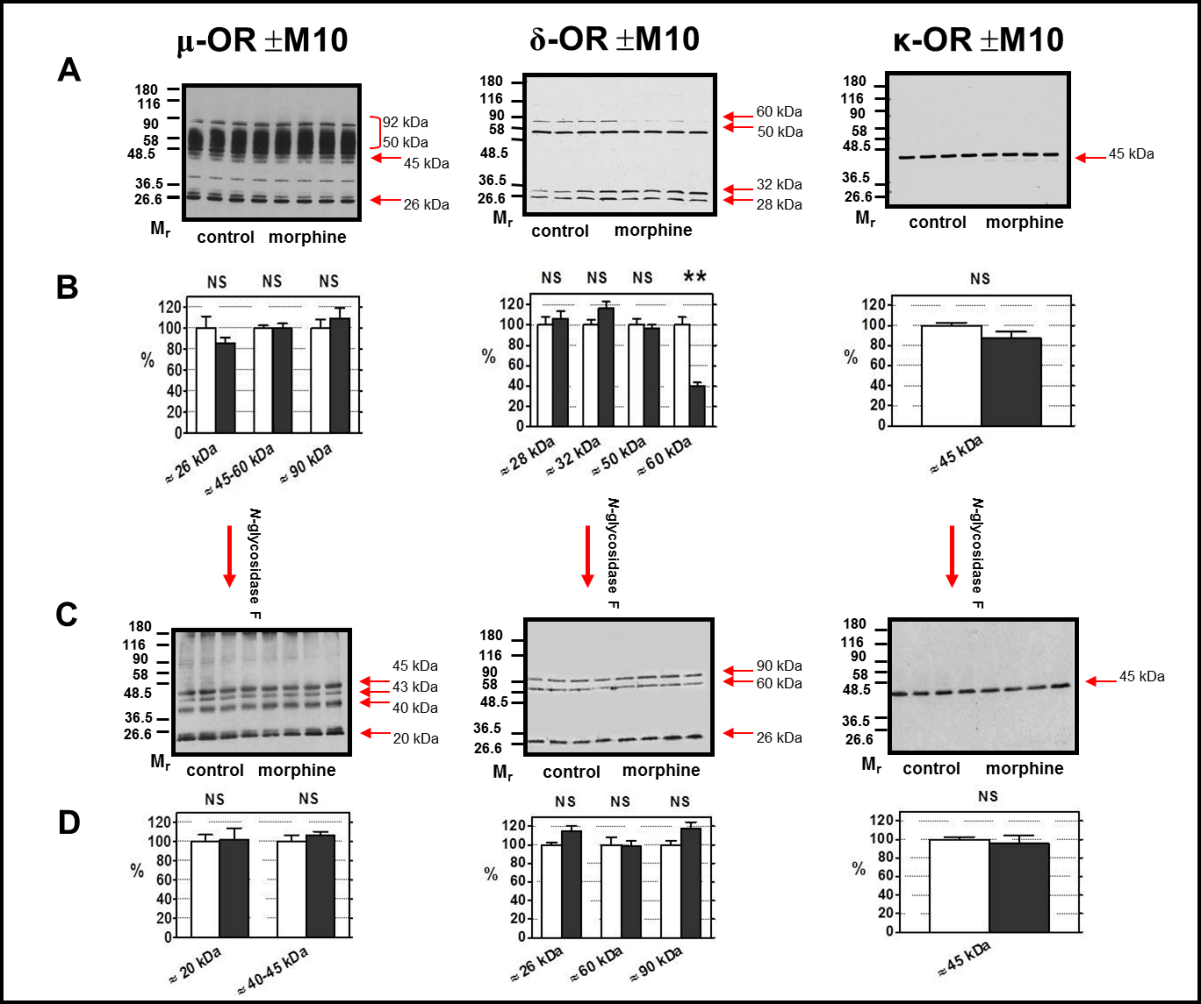
Detection of μ-OR, δ-OR and κ-OR in PNS fractions (±M10) treated with *N*-glycosidase F. PNS fractions prepared from FBC of rats in groups (+M10) and (─M10) were treated with SDS, Triton X-100 and *N*-glycosidase F (NGF) as described in Methods. 20 μg of protein was applied per each lane of 10% w/v acrylamide/0.26% w/v bis-acrylamide gels (four + four) and resolved under the dissociated (+DTT) conditions. The OR were recognized by antibodies from Santa Cruz: μ-OR (C-20, sc-7488-R, C-terminus), δ-OR (H-60, sc-9111, N-terminus), and κ-OR (H-70, sc-9112, N-terminus). Results represent the typical immunoblots of NGF-untreated **(A)** and NGF-treated **(C)** samples. The significance of difference between NGF-untreated (+M10) versus (─M10) PNS samples **(B)** and NGF-treated (+M10) versus (─M10) PNS samples **(D)** was analyzed by Student´s *t*-test using GraphPad*Prizm4* (NS, p>0.05). The results represent the average signal of three immunoblots, each performed with four control + four morphine-treated samples of PNS ± SEM. 100% on y-axis (lower panels) represents the average intensity of a given immunoblot signal determined in PNS prepared from control, (─M10) rats.

In the next step of our work, we tested the role of *N*-glycosylation in detection of OR in rat FBC as a multiplicity of positive signals recognized by various antibodies may be primarily caused by the heavy glycosylation of N-terminus of OR proteins. The (+M10) and (─M10) samples of PNS were exposed to Triton X-100 and *N*-glycosidase F (NGF) as described in Methods and μ-OR determined in the same way as before with Ab C-20. The strong signals visible in 50–90 kDa area of the immunoblots (**Fig 6,** upper left panel) were completely missing in *N*-glycosidase-treated samples (**Fig 6,** lower left panel). The remaining signals were represented by four bands with M_w_ of ≈ 45 kDa, ≈ 43 kDa, ≈ 40 kDa and ≈ 20 kDa.

This type of alteration of immunoblot profiles of μ-OR by *N*-glycosidase F was close if not identical with the out-come of studies of μ-OR detection by C-terminus oriented Ab in mouse or rat neuronal tissues by Huang and Liu-Chen [25]. They detected the broad and diffuse band in M_w_ range between 58 to 97 kDa with the median M_w_ above 62 kDa (depending on brain regions and species). The broad and diffuse feature of this protein band indicated glycosylation of μ-OR. After treatment with PNGase F, μ-ORs became sharp bands with molecular weights close to the theoretical molecular mass (~43 kDa) of the deduced amino acid sequences [25].

Comparison of (+M10) and (─M10) PNS samples treated with Triton X-100 and NGF (**Fig 6**, lower left panel) indicated that none of μ-OR protein bands was significantly altered by morphine, p > 0.05.

The immunoblot profile of δ-OR obtained with N-terminus-oriented Ab H-60 (**Fig 6,** upper middle panel) was also substantially altered by NGF. The “new” signal was noticed at M_w_ ≈ 90 kDa (**Fig 6**, lower middle panel) and this change was accompanied by disappearance of δ-OR protein with M_w_ ≈ 50 kDa. The intensity of immunoblot signal detected at M_w_ ≈ 60 kDa was not significantly different when compared in control and morphine-treated samples of PNS.

Formation of ≈ 90 kDa protein, the disappearance of the signal at ≈ 50 kDa and lack of morphine effect on ≈ 60 kDa isoform of δ-OR, observed after combined action of 0.2% (w/v) SDS, 1.5% (v/v) Triton X-100 and NGF on immunoblot profile of δ-OR in rat brain PNS, represent the set of similar observations as those described before by Cvejic and Devi [27] and Devi [28] in CHO cells stably expressing Flag-epitope tagged version of δ-OR. The NGF-treatment of these cells resulted in formation of “new protein” signal exhibiting M_w_ of ≈ 80 kDa. This protein was regarded to represent a deglycosylated dimmer of Flag-δ-OR.

The single, strong signal of κ-OR detected by Ab H-70 at ≈ 45 kDa was not effected by NGF treatment and unchanged by morphine (**Fig 6,** right panels).

**Fig 7:**
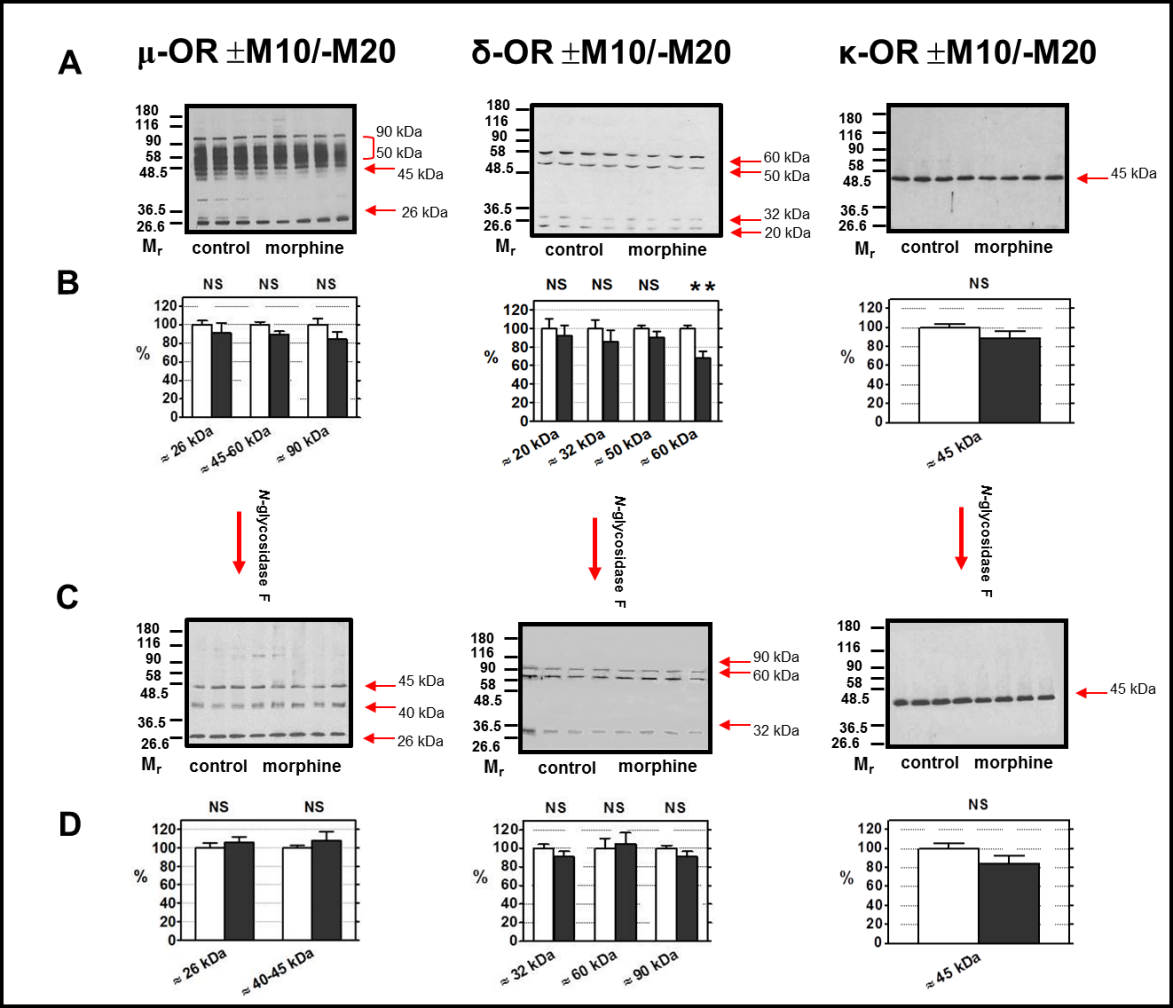
Detection of μ-OR, δ-OR and κ-OR in PNS fractions (±M10/─M20) treated with *N*-glycosidase F. PNS fractions prepared from FBC of rats in groups (+M10/─M20) and (— M10/─M20) were treated with SDS, Triton X-100 and *N*-glycosidase F (NGF) as described in Methods. 20 μg of protein was applied per each lane of 10% w/v acrylamide/0.26% w/v bis-acrylamide gels (four + four) and resolved under dissociated (+DTT) conditions. The OR were recognized by antibodies from Santa Cruz: μ-OR (C-20, sc-7488-R, C-terminus), δ-OR (H-60, sc-9111, N-terminus), and κ-OR (H-70, sc-9112, N-terminus). Results represent the typical immunoblots of NFG-untreated **(A)** and NGF-treated **(C)** samples. The significance of difference between NGF-untreated (+M10/─M20) versus (─M10/─M20) PNS samples **(B)** and NGF-treated (+M10/─M20) versus (─M10/─M20) PNS samples **(D)** was analyzed by Student´s *t*-test using GraphPad*Prizm4* (NS, p>0.05). The results represent the average signal of three immunoblots, each performed with four control + four morphine-treated samples of PNS ± SEM. 100% on y-axis (lower panels) represents the average intensity of a given immunoblot signal determined in PNS prepared from control, (─M10) rats.

Virtually the same results were obtained when testing the NGF effect on (+M10/─M20) and (─M10/─M20) samples of PNS. The strong signals of μ-OR recognized by Ab C-20 at M_w_ >50 kDa were absent in NGF-treated samples (**Fig 7**, left panels), δ-OR protein with M_w_ ≈ 90 kDa was clearly manifested in both types of PNS samples, the ≈ 60 kDa isoform of δ-OR was unchanged (**Fig 7**, middle panels) and the single, strong signal of κ-OR produced by Ab H-70 at ≈ 45 kDa was not effected by neither NGF nor morphine (**Fig 7,** right panels).

### Immunoblot detection of μ-OR, δ-OR and κ-OR in 2D gels

As mentioned in introduction, our previous analysis of rat brain plasma membrane-enriched fractions by 2D-ELFO and LC-MS/MS [17] distinguished the morphine-responsive pool of trimeric Gβ subunits that decreased 2-fold with morphine administration. This pool of Gβ subunits represented just a *minor* fraction of the total pool of Gβ subunits that was unchanged by morphine. Hence, in our present work, we also tested the efficiency of 2D-ELFO combined with immunoblotting for resolution of different isoforms of μ-OR, δ-OR, and κ-OR, and compared the control and morphine-treated PNS samples.

In control PNS samples (**S4 Fig**, left panels), μ-OR-oriented Ab C-20 recognized the two strong signals at pI ≈ 5.2 and pI ≈ 6.7–7.4 (M_w_ ≈ 40–45 kDa). The third μ-OR protein with M_w_ ≈ 26.6–37 kDa was detected in the alkaline region of the 2D gels at pI ≈ 9.8. These immunoblot signals were also detected in morphine-treated samples of PNS (**S4 Fig**, right panels). Immunoblot detection of δ-OR in 2D gels (**S5 Fig**) led to the recognition of five distinct immunoblot signals observed across a wide range of pI (from 5 to 10), but the position and shape of these spots was not the same for the control versus morphine-treated PNS samples. A similar situation was observed when the distribution of κ-OR immunoblot signals in 2D gels was tested with Ab H-70 (**S6 Fig**). Obviously, the heterogeneities in position and number of the resolved proteins when comparing control and morphine-treated PNS samples precluded any effort to make a quantitative analysis of 2D immunoblots.

Intensive removal of lipids by acetone, degradation of hydrophobic bonds by highly concentrated urea (7M) plus thiourea (2M), reduction of–SH groups by DTT and methylation by iodoacetamide, all these steps in preparation of PNS samples for 2D-ELFO, represent the explanation why a substantial difference between immunoblot profiles of OR resolved by 1D- and 2D-SDS-PAGE was observed.

### Na, K-ATPase, caveolin-1, actin and glyceraldehyde 3P-dehydrogenase (GAPDH) in FBC of rats exposed to morphine for 10 days; comparison with animals sacrificed 20 days since morphine withdrawal

**Fig 8:**
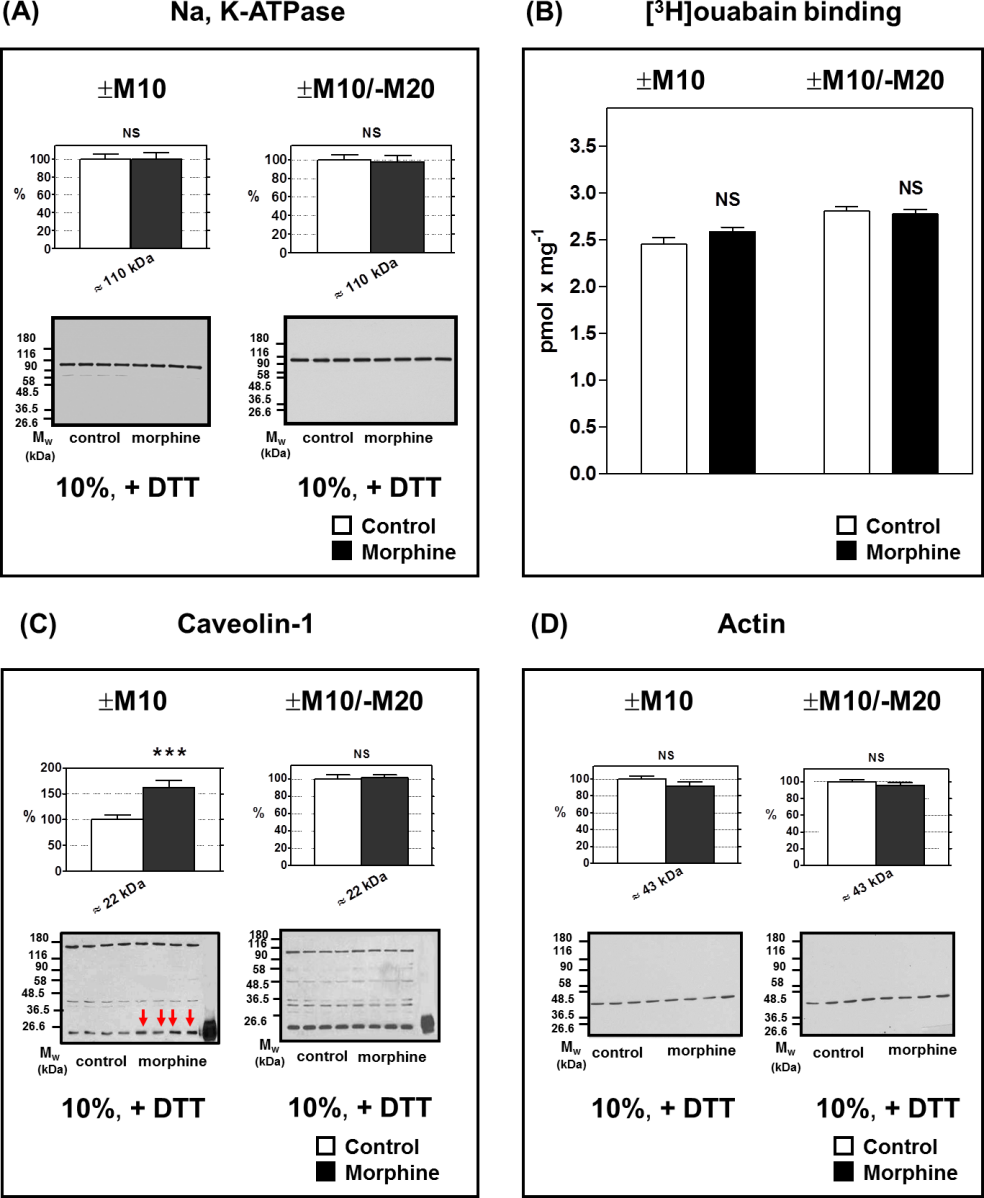
Na, K-ATPase, caveolin-1 and actin in PNS fractions preparad from experimental groups (±M10) and (±M10/─M20). Na, K-ATPase **(A),** [^3^H]ouabain binding **(B),** caveolin-1 **(C)** and actin **(D)** levels were determined in (+M10), (─M10), (+M10/─M20) and (─M10/─M20) samples of PNS. PNS proteins (20 μg per lane) were resolved under dissociated conditions (+DTT) by standard SDS-PAGE in 10% w/v acrylamide/0.26% w/v bis-acrylamide gels, and immunoblotted. Antibodies H-300 (sc-28800) and I-19 (sc-1616) from Santa Cruz were used for recognition of α subunit of Na, K-ATPase and actin, respectively. [^3^H]ouabain binding assay was performed at saturating, 18 nM concentration as described in Methods. Caveolin-1 (↓) was identified by Ab C13630-050 from Transduction Laboratories. The human endothelial cell lysate was used as a positive control for caveolin-1 (Transduction Laboratories, in last lane of immunoblots). Results represent the average of three immunoblots of Na, K-ATPase or [^3^H]ouabain binding assays ± SEM, each performed in quadruplicates (upper columns). Analysis of caveolin-1 and actin was based on signals collected from five and three immunoblots, each performed with four control + four morphine-treated samples of PNS, respectively. 100% on y-axis (upper panels) represents the average intensity of a given immunoblot signal determined in PNS prepared from control, (─M10) rats. The significance of difference between the control and morphine-treated samples was analyzed by Student´s *t*-test using GraphPad*Prizm4*. In the lower panels, typical immunoblots are shown (NS, p > 0.05; ***, p < 0.001).

The last part of our work tested the specificity of morphine effects. In parallel PNS samples to those utilized for determination of opioid receptors, the content of Na, K-ATPase was measured as a negative standard of morphine action and a prototypical PM marker which would not have been expected to be altered by morphine treatment [6,10]. Quantitative analysis of the immunoblot signals corresponding to the α subunit of Na, K-ATPase indicated that the expression level of this protein in FBC was not modified by exposure of rats to increasing doses of morphine for 10 days (**Fig 8A**, left panel; ±M10). Accordingly, Na, K-ATPase level in PNS samples prepared from rats sacrificed 20 days since their last dose of morphine was the same as in corresponding controls (**Fig 8A**, right panel; ±M10/─M20). These findings were verified by radioligand-binding assays featuring the binding of selective inhibitor of this enzyme—the number of specific [^3^H]ouabain sites at saturating, 18 nM concentration was not significantly different when compared in (+M10) and (─M10) samples of PNS (**Fig 8B**, left panel); the same result was obtained in PNS prepared from morphine-treated rats after 20 days of morphine withdrawal (**Fig 8B**, right panel; ±M10/─M20).

Our previous work, which was methodologically based on 2D-ELFO and proteomic analysis indicated the up-regulation of proteins functionally related to *oxidative stress* and *apoptosis* [17]. We have also shown that the decrease of cholesterol level in HEK293 cells stably expressing δ-OR did not alter the agonist-binding site of δ-OR (B_max_ and K_d_), but the ability of δ-OR agonist DADLE to activate G proteins was markedly impaired—EC_50_ values of DADLE-stimulated [^35^S]GTPγS binding was shifted by one order of magnitude to the right [29]. The alteration of PM integrity by partial cholesterol depletion further led to a decrease of DADLE-induced δ-OR internalization [30]. The cholesterol depletion also resulted in decrease of efficiency of the TRH-R-initiated signaling cascade [31–33] and slowing of mobility of TRH-R in live HEK293 cells [34].

Based on these results and considering the previously published evidence for participation of cholesterol-rich membrane domains (MD) in OR signaling [35–41], we have also tested the morphine-induced change of MD marker caveolin-1 and cholesterol level.

We found that caveolin-1 was significantly up-regulated in brains of rats exposed to morphine for 10 days, ***, p<0.001, (group +M10; **Fig 8C,** left panels). Of note is that this increase disappeared after 20 days of morphine withdrawal (group +M10/─M20; **Fig 8C**, right panels). Actin levels, which were measured in the context of eventual *drastic* alteration of brain cell structure, were not affected by morphine in (+M10) or (+M10/─M20) PNS samples when compared with corresponding controls (**Fig 8D**). Determination of another “negative control” of morphine action, glyceraldehyde-3P-dehydrogenase (**S7 Fig**), did not indicate any change in expression level of this important regulatory enzyme of intracellular energy metabolism in FBC.

Reversible up-regulation of caveolin-1 may be therefore regarded as an indicator of temporary brain cell “discomfort” or “stress” observed after exposure of experimental animals to morphine for 10 days. Interestingly, the amount of cholesterol in morphine-treated samples of PNS (45.0 ± 0.7 μg/mg protein) was significantly higher than in controls (40.6 ± 0.4 μg/mg protein, ***, p<0.001) and was reversed back to control levels after 20 days of abstinence (**Table 1**). Thus, cholesterol content followed a similar pattern as morphine-induced alteration of caveolin-1 level.

**Table 1:** Cholesterol amount in PNS prepared from FBC of control (─M10) and morphine-treated rats (+M10); comparison with animals sacrificed 20 days since the morphine withdrawal (±M10/─M20).

PNS sample	μg/mg protein	%	p
**-M10**	40.6 ± 0.4	100 ± 1	
**+M10**	45.0 ± 0.7	111 ± 2	<0.001, ***
**(-M10/-M20)**	42.2 ± 0.4	100 ± 1	
**(+M10/-M20)**	42.0 ± 0.5	100 ± 1	>0.05, NS^a^

Data represent the average of 3 experiments ± SEM.

***High significant difference, p<0.001.

^a^ Not significant difference, p>0.05.

## Discussion

Content of μ-OR in the mammalian brain was determined by numerous methods, including Northern blot analysis, RNase protection assays, RT-PCR, immunohistochemistry and specific radioligands [22, 42–44]. However, the number of reports using immunoblot analysis for determination of μ-OR protein content in FBC was rather limited and vastly heterogeneous values of M_w_ of μ-OR-related proteins recognized by specific antibodies were described. There were two major trends in results arrived at: broad and diffuse bands with higher M_w_ vs. sharp bands with lower M_w_. A number of researchers identified μ-OR as sharp bands with M_w_ values between 40 kDa and 60 kDa in mouse or rat neuronal extracts [24,45]; in contrast, other researchers demonstrated μ-OR as a single, diffuse band with a M_w_ between 58 kDa and 97 kDa [25]. When distinguished by C-terminus-oriented Ab and upon deglycosylation, μ-OR in rat caudate putamen and thalamus was observed as a single sharp band with a M_w_ close to the theoretical molecular mass (≈ 43 kDa) deduced from the amino acid sequence of this receptor.

The single, strong immunoblot signal for μ-OR exhibiting a M_w_ of ≈ 60 kDa was detected by the C-terminus-oriented Ab, C-20, in morphine-treated samples of PNS resolved by 1D-SDS-PAGE in 10% acrylamide gels as well as 4–12% NuPAGE gels (**Fig 1A** and **1B**) in the absence of DTT (─DTT). The average intensity of this signal was not significantly different when compared to (+M10) and (─M10) samples of PNS. Resolution of PNS proteins under dissociated conditions (+DTT) revealed the presence of multiple protein bands exhibiting a wide range of M_w_ ≈ 20–90 kDa (**Fig 1C** and **1D**). The total signal of all these proteins was unchanged by morphine. Therefore, when tested under both non-reducing and reducing conditions and in different gels, prolonged exposure of rats to high doses of morphine for 10 days was without effect on μ-OR level. The same result was obtained in PNS prepared from rats exposed to morphine for 10 days and subsequently nurtured for 20 days without it (**Fig 4,** left panels).

Our experience with detection of μ-OR in deglycosylated samples of PNS was similar to that reported by Huang and Liu-Chen [25]—the strong signals of μ-OR with M_w_ ≈ 50–90 kDa (**Fig 6**, upper left panel) were not observed in PNS samples treated with SDS, Triton X-100 and NGF (**Fig 6,** lower left panel); the remaining signals were represented by four distinct protein bands with M_w_ values of ≈ 20 kDa, ≈ 40 kDa, ≈ 43 kDa and ≈ 45 kDa (**Fig 6,** lower left panel). Basically the same result was observed in (±M10/─M20) PNS samples **(Fig 7,** lower left panel). None of these μ-OR-related immunoblot signals were altered when compared in control and morphine-treated samples of PNS.

From a clinical point of view, the drug withdrawal is the most powerful factor driving opioid dependence and addictive behavior [46] and represents the traumatic event with severely adverse effects, such as sedation, respiratory depression, cramps, changes in body temperature, diarrhoea, and emesis [47,48]. Evidently, prolonged abstinence is associated with continuous behavioral and neurobiological abnormalities that are responsible for drug-seeking reinstatement [49,50]. Recent data of Erbs et al. [51] using δ-OR-eGFP knock-in mouse indicated that application of increasingly escalated doses of morphine for 5 days (from 20 to 100 mg/kg i. p.) resulted in a decrease of density of δ-OR-GFP-expressing neurons in the mouse dorsal hippocampus; this change persisted after four weeks of abstinence. The decrease of δ-OR-positive cell density in specific areas of the hippocampus was linked to redistribution of these receptors from the cell interior to the plasma membrane.

In our experiments dealing with FBC, the δ-OR isoform with M_w_ ≈ 60 kDa was significantly decreased in (+M10) samples of PNS (**Fig 2**) and, importantly, when considering the data of Erbst et al. [51], this decrease persisted for 3 weeks of abstinence, i.e., it was also detected in ±M10/─M20 samples of PNS (**Fig 4**, middle panels). Intensity of other immunoblot signals of δ-OR, i.e. those detected at ≈ 28 kDa, ≈ 32 kDa and ≈ 50 kDa, was not altered by morphine and the situation remained this way after 20 days of drug withdrawal. The total signal of all four isoforms of δ-OR proteins in (+M10) samples of PNS was decreased to 87 ± 6%, but this decrease was not significant. The same applied for total signal of δ-ORs in PNS prepared from (+M10/─M20) group of rats.

κ-OR, the third type of OR, represents an important participant in the regulation of both reward and mood processes [52] and modulates many physiological functions, such as pain, drinking, water balance, food intake, control of salt appetite, temperature control, and various endocrine functions [53,54]. Activation of κ-OR opposes μ-OR in the regulation of “hedonic” homeostasis. The preclinical evidence supports the view that κ-OR blockade may beneficially alleviate stress responses, reduce drug craving, and remediate depressive states [55–57].

Detection of κ-OR in FBC of control and morphine-treated rats was by far more straightforward than that of μ-OR and δ-OR (**Fig 3**). Resolution of PNS proteins under non-dissociated (— DTT) and dissociated (+DTT) conditions and in 10% w/v acrylamide/0.26% w/v bis-acrylamide as well as 4–12% gradient gels uncovered the presence of the single, strong signal of this receptor protein with M_w_ ≈ 45 kDa that was not altered by morphine. The same was found in rats after 20 days of morphine withdrawal (**Fig 4**, right panels).

Data obtained by immunoblot analysis (**Figs 1–4**), namely the lack of morphine effect on the total level of μ-, δ- and κ-OR, were supported by radioligand binding assays using the non-specific radioligands [^3^H]naloxon and [^3^H]diprenorphine (**Fig 5**). Results presented in **Fig 5A** indicated that the number of μ+δ+κ-OR binding sites for both these radioligands was not altered by morphine in (±M10) as well as (±M10/─M20) samples of PNS. The same results were obtained by analysis of PM fractions isolated from FBC (**Fig 5B).**

The prototypical plasma membrane marker, Na, K-ATPase, which in our experiments was measured as a negative standard of morphine action, was unchanged when compared in groups (+M10) and (─M10) or (+M10/─M20) and (─M10/─M20) of the experimental animals (**Fig 8A** and **8B**). This supports the view that the morphine-induced change in plasma membrane structure and function does not denote a drastic event requiring alterations in the expression of the enzyme responsible for homeostasis of the major brain electrolytes, sodium and potassium cations. Accordingly, the level of actin which was measured as a test of eventual change of overall brain cells structure, was not affected by morphine in (±M10) as well as (±M10/─M20) PNS samples (**Fig 8D**). The same negative result was obtained by determination of GAPDH level, an indicator of alteration of intracellular FBC energy metabolism (**S7 Fig**).

Caveolin-1 was in our study measured as a marker of PM compartments denominated as membrane domains/lipid rafts characterized by large amounts of cholesterol, sphingolipids, and trimeric G proteins [58,59]. These PM compartments participate in regulation of signaling cascades initiated by many GPCRs [60,61], opioid receptors included [37–39]. Membrane domains also fulfill an important role in maintaining cholesterol balance of mammalian organism by mediating cholesterol transport from intracellular organelles to the cell surface and extracellular cholesterol acceptors.

Of particular interest was that in our present experiments, the highly significant up-regulation of caveolin-1 was noticed in (+M10) PNS samples and this elevation disappeared after 20 days of morphine withdrawal (**Fig 8C**). The same was seen when measuring cholesterol concentrations, which were increased after 10 days with morphine and returned back to control levels after 20 days of morphine withdrawal (**Table 1**). The up-regulation of caveolin-1 and cholesterol is in line with our recent data [16] indicating that FBC of rats exposed to morphine for 10 days is substantially altered as far as the overall protein composition is concerned. Number of altered proteins identified by proteomic analysis was decreased from 28 to 14 (MALDI-TOF MS/MS) or from 113 to 19 (MaxLFQ), respectively. Thus, a mammalian organism exhibits a great ability to oppose the morphine-induced changes in the target tissue protein composition with the aim of returning to physiological norm subsequently to drug withdrawal.

## Conclusions

1. This work presents the detailed immunoblot analysis of μ-, δ- and κ-OR proteins in frontal brain cortex of rats (FBC) exposed to morphine for 10 days (groups ±M10) or, exposed to morphine and subsequently nurtured for 20 days without this drug (groups ±M10/−M20).
2. PNS fraction was prepared from FBC homogenate, resolved by SDS-PAGE in 10% and 4–12% gradient gels under non-dissociated (─DTT) or dissociated (+DTT) conditions and analyzed for the content of μ-, δ- and κ-OR by antibodies oriented against C- and N-terminus of these receptors.
3. The total number of μ+δ+κ-OR was also determined by radioligand binding assays using non-selective antagonist [^3^H]naloxon and agonist [^3^H]diprenorphine.
4. The ≈ 60 kDa form of δ-OR was significantly decreased in morphine-treated rats; this decrease was also noticed in PNS samples prepared from rats after 20 days of the drug withdrawal. Immunoblot signals of other forms of μ-, δ- and κ-opioid receptors were not altered by morphine.
5. Plasma membrane marker, Na, K-ATPase, cytoskeleton marker actin and intracellular energy metabolism marker GAPDH, which were determined as negative standards of morphine action, were unaffected.
6. Noticeably, the highly significant elevation of caveolin-1 and cholesterol level was measured in FBC of morphine-treated rats and this increase returned back to control levels after 20 days of morphine withdrawal.
7. It may be suggested that the reversible up-regulation of caveolin-1 is a consequence of the rise in brain cell cholesterol level and MD/lipid rafts participate in morphine-induced alteration of brain cell metabolism.

## Supporting information

S1 Fig Specifity of antibodies used for determination of μ-OR. The immunoblot profile of Ab C-20 (sc-7488-R, C-terminus) from Santa Cruz as presented in datasheet of supplier.
(TIF)

S2 Fig Immunoblot detection of δ-OR in HEK293 cells stably expressing Flag-δ-OR. PNS fraction was prepared from HEK293 cells stably expressing Flag-δ-OR as described before by Brejchova et al. [34], exactly the same amount of protein (20 μg) applied per each lane, resolved under dissociated conditions (+DTT) by standard SDS-PAGE in 10% acrylamide /0.26% bis-acrylamide gel and immunoblotted with N-terminus-oriented Ab H-60 (sc-9111) from Santa Cruz.
(TIF)

S3 Fig Specifity of antibodies used for determination of κ-OR. The immunoblot profile of Ab H-70 (sc-9112, N-terminus) from Santa Cruz as presented in datasheet of supplier.
(TIF)

S4 Fig Resolution of μ-OR isoforms by 2D-ELFO. Immunoblot detection of μ-OR in 2D gels. **(A)** The (+M10) and (─M10) or **(B)** (+M10/─M20) and (─M10/─M20) samples of PNS (2 mg protein per gel) were extracted in acetone/TCA and resolved by 2D-ELFO as described in Methods. The μ-OR was recognized by Ab C-20 (sc-7488-R). The two immunoblot signals with similar M_w_ of 40–45 kDa were observed at pI ≈ 5.2 and pI ≈ 6.7–7.4, respectively. The third protein signal of M_w_ ≈ 26.6–37 kDa was detected in alkaline area of 2D gels at pI ≈ 9.8.
(TIF)

S5 Fig Resolution of δ-OR isoforms by 2D-ELFO. Immunoblot detection of δ-OR in 2D gels. **(A)** The (+M10) and (─M10) or **(B)** (+M10/─M20) and (─M10/─M20) samples of PNS (2 mg protein per gel) were extracted in acetone/TCA and resolved by 2D-ELFO as described in Methods. The δ-OR was recognized by Ab H-60 (sc-9111). Five distinct immunoblot signals were observed in a wide range of pI varying from 5 to 10.
(TIF)

S6 Fig Resolution of κ-OR isoforms by 2D-ELFO. Immunoblot detection of κ-OR in 2D gels. **(A)** The (+M10) and (─M10) or **(B)** (+M10/─M20) and (─M10/─M20) samples of PNS (2 mg protein per gel) were extracted in acetone/TCA and resolved by 2D-ELFO as described in Methods. The κ-OR was recognized by Ab H-70 (sc-9112).
(TIF)

S7 Fig Determination of GAPDH content in PNS fractions prepared from experimental groups (±M10) and (±M10/─M20). PNS proteins (20 μg per lane) were resolved under dissociated conditions (+DTT) by standard SDS-PAGE in 10% acrylamide/0.26% bis-acrylamide gel and immunoblotted. Antibodies FL-335 from Santa Cruz were used for detection of GAPDH. Statistical analysis was based on signals collected from three immunoblots, each performed with four control + four morphine-treated samples of PNS, respectively. 100% on y-axis (upper panels) represents the average intensity of a given immunoblot signal determined in PNS prepared from control, (─M10) rats. Significance of difference between the control and morphine-treated samples was analyzed by Student´s *t*-test using GraphPad*Prizm4*. In the lower panels, typical immunoblots are shown. NS, p>0.05.
(TIF)

S1 File List of abbreviations. (DOCX)

S2 File The ARRIVE guidelines checklist. (PDF)
