# Supplementary figures and images for: Determination of μ-, δ- and κ-opioid receptors in forebrain cortex of rats exposed to morphine for 10 days: Comparison with animals after 20 days of morphine withdrawal
Source: PLoS One. 2017 Oct 20;12(10):e0186797. doi: 10.1371/journal.pone.0186797 (PMC5650167; doi:10.1371/journal.pone.0186797)

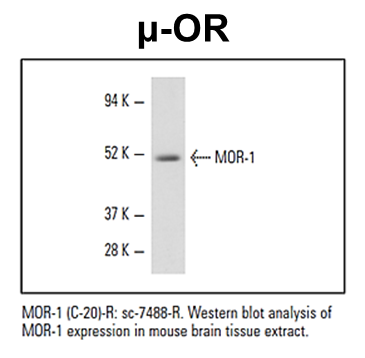

Supplement: S1 Fig — The immunoblot profile of Ab C-20 (sc-7488-R, C-terminus) from Santa Cruz as presented in datasheet of supplier. (TIF) [file pone.0186797.s001.tif]

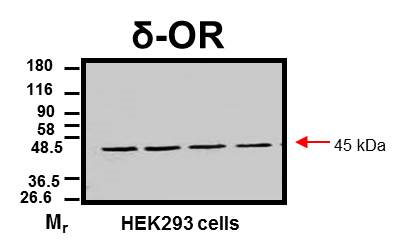

Supplement: S2 Fig — PNS fraction was prepared from HEK293 cells stably expressing Flag-δ-OR as described before by Brejchova et al. [34], exactly the same amount of protein (20 μg) applied per each lane, resolved under dissociated conditions (+DTT) by standard SDS-PAGE in 10% acrylamide /0.26% bis-acrylamide gel and immunoblotted with N-terminus-oriented Ab H-60 (sc-9111) from Santa Cruz. (TIF) [file pone.0186797.s002.tif]

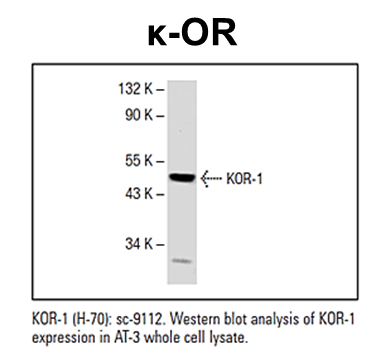

Supplement: S3 Fig — The immunoblot profile of Ab H-70 (sc-9112, N-terminus) from Santa Cruz as presented in datasheet of supplier. (TIF) [file pone.0186797.s003.tif]

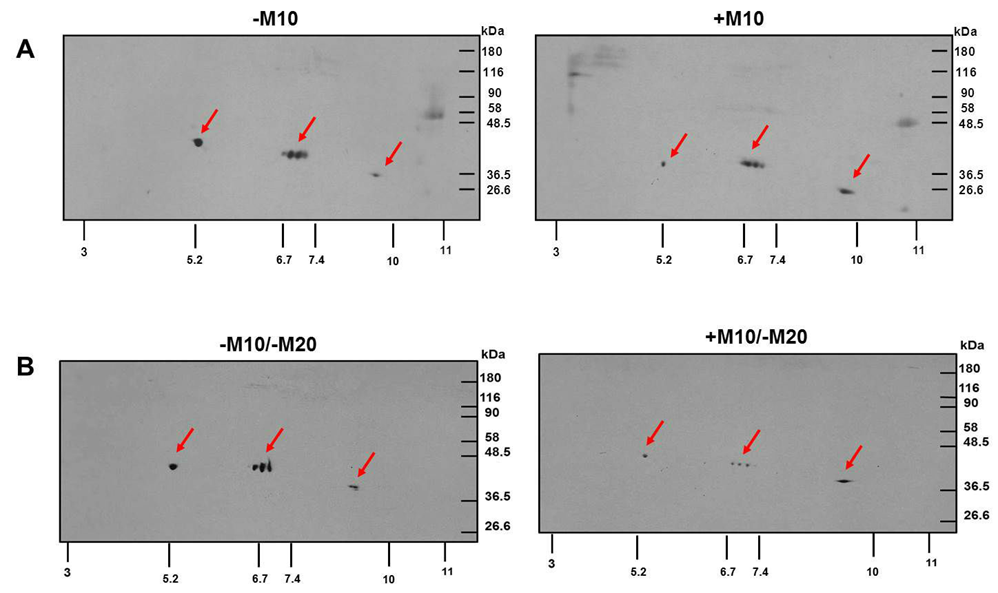

Supplement: S4 Fig — Immunoblot detection of μ-OR in 2D gels. (A) The (+M10) and (─M10) or (B) (+M10/─M20) and (─M10/─M20) samples of PNS (2 mg protein per gel) were extracted in acetone/TCA and resolved by 2D-ELFO as described in Methods. The μ-OR was recognized by Ab C-20 (sc-7488-R). The two immunoblot signals with similar Mw of 40–45 kDa were observed at pI ≈ 5.2 and pI ≈ 6.7–7.4, respectively. The third protein signal of Mw ≈ 26.6–37 kDa was detected in alkaline area of 2D gels at pI ≈ 9.8. (TIF) [file pone.0186797.s004.tif]

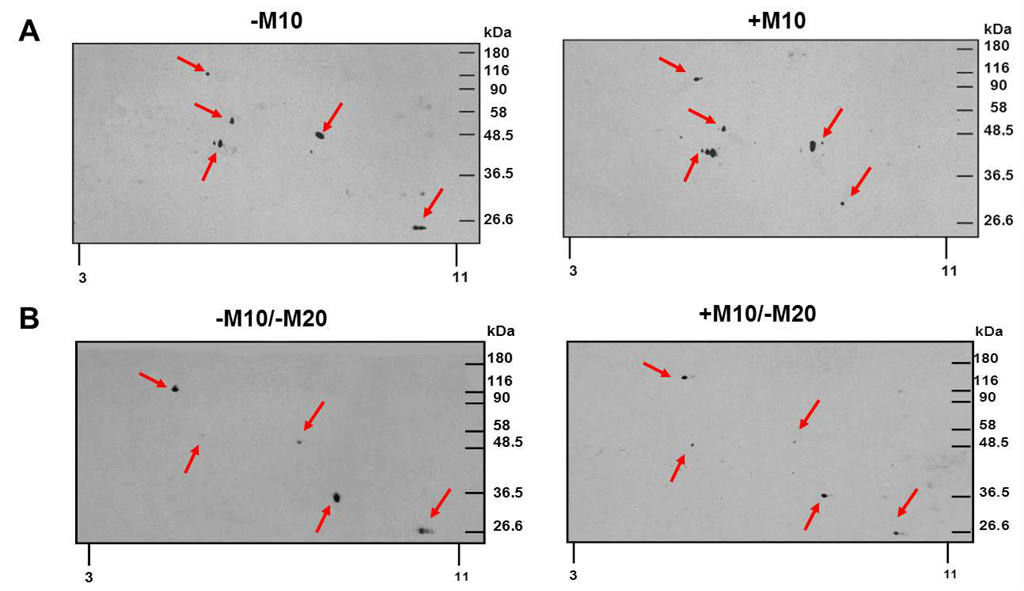

Supplement: S5 Fig — Immunoblot detection of δ-OR in 2D gels. (A) The (+M10) and (─M10) or (B) (+M10/─M20) and (─M10/─M20) samples of PNS (2 mg protein per gel) were extracted in acetone/TCA and resolved by 2D-ELFO as described in Methods. The δ-OR was recognized by Ab H-60 (sc-9111). Five distinct immunoblot signals were observed in a wide range of pI varying from 5 to 10. (TIF) [file pone.0186797.s005.tif]

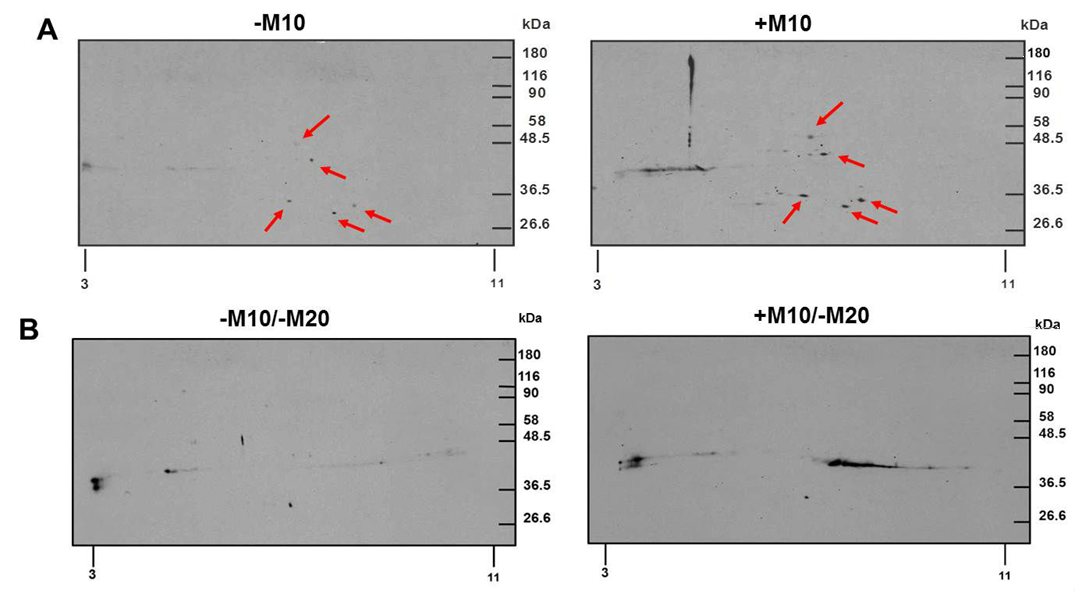

Supplement: S6 Fig — Immunoblot detection of κ-OR in 2D gels. (A) The (+M10) and (─M10) or (B) (+M10/─M20) and (─M10/─M20) samples of PNS (2 mg protein per gel) were extracted in acetone/TCA and resolved by 2D-ELFO as described in Methods. The κ-OR was recognized by Ab H-70 (sc-9112). (TIF) [file pone.0186797.s006.tif]

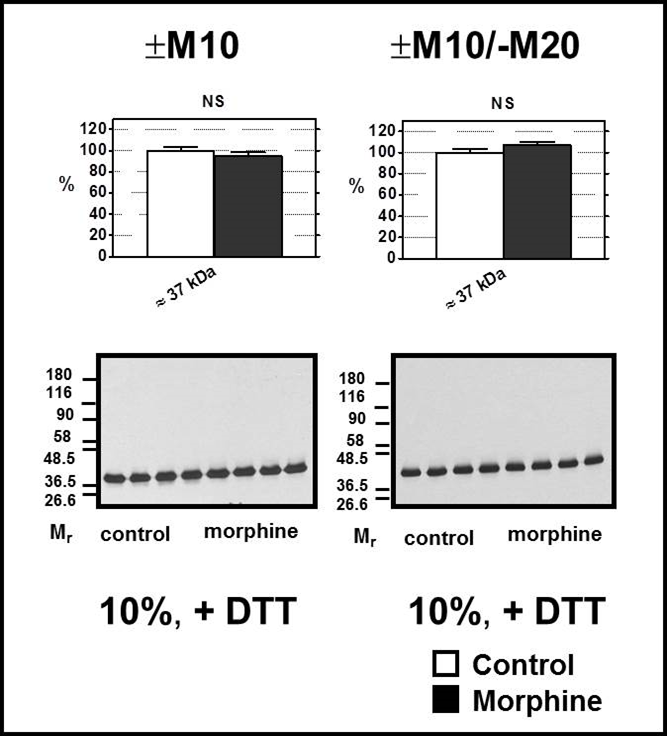

Supplement: S7 Fig — PNS proteins (20 μg per lane) were resolved under dissociated conditions (+DTT) by standard SDS-PAGE in 10% acrylamide/0.26% bis-acrylamide gel and immunoblotted. Antibodies FL-335 from Santa Cruz were used for detection of GAPDH. Statistical analysis was based on signals collected from three immunoblots, each performed with four control + four morphine-treated samples of PNS, respectively. 100% on y-axis (upper panels) represents the average intensity of a given immunoblot signal determined in PNS prepared from control, (─M10) rats. Significance of difference between the control and morphine-treated samples was analyzed by Student´s t-test using GraphPadPrizm4. In the lower panels, typical immunoblots are shown. NS, p>0.05. (TIF) [file pone.0186797.s007.tif]
