# Supplementary material for: Determination of μ-, δ- and κ-opioid receptors in forebrain cortex of rats exposed to morphine for 10 days: Comparison with animals after 20 days of morphine withdrawal
Source: PLoS One. 2017 Oct 20;12(10):e0186797. doi: 10.1371/journal.pone.0186797 (PMC5650167; doi:10.1371/journal.pone.0186797)
Supplement: S1 File — (DOCX) [file pone.0186797.s008.docx]

**Abbreviations:** Ab, antibodies**;** AC, adenylyl cyclase; BPB, bromophenol blue; B_sp_, specific binding; B_t_ , total binding; B_nsp_, non-specific radioligand binding; cAMP, cyclic 3´, 5´, -adenosine monophosphate; CBB, Colloidal Coomassie Blue; DAMGO, [(2-D-alanine2-4-methylphenylalanine-5-glycineol)-enkefalin, Tyr-D-Ala-Gly-N-methyl-Phe-Gly-ol]; DADLE, [(2-D-alanine-5-D-leucine)-enkefalin, Tyr-D-Ala-Gly-Phe-D-Leu]; δ-OR, -opioid receptor; DTT, dithiothreitol; EDTA, ethylenediamine-tetraacetic acid; 2D-ELFO, two-dimensional gel electrophoresis; FBC, frontal brain cortex /forebrain cortex; GAPDH, glyceraldehyde-3P-dehydrogenase; GPCR, G-protein-coupled receptor; G proteins, heterotrimeric guanine nucleotide-binding regulatory proteins; h, hour; CHAPS, 3-[(3-cholamidopropyl) dimethylammonio]-1-propanesulfonate; IEF, isoelectric focusing; κ-OR, κ-opioid receptor; LC-MS/MS, liquid chromatography-mass spectrometry; LFQ, label-free quantification; (+M10), rats exposed to increasing doses (10-40 mg/kg) of morphine for 10 days; (─M10); control rats injected with saline for 10 days; (+M10/─M20), rats exposed to morphine for 10 days and subsequently nurtured for 20 days without drug; (─M10/─M20), rats injected with saline for 10 days and subsequently nurtured for 20 days without drug; MALDI-TOF MS/MS, Matrix-assisted laser desorption/ionization time-of-flight mass spectrometry; MD, membrane domains; MOPS, 3-(N-morpholino) propane sulfonic acid; μ-OR, µ-opioid receptor; M_w_, molecular mass/weight; NGF, *N*-glycosidase F; Na, K-ATPase, sodium plus potassium, ouabain-dependent adenosine triphosphatase (EC 3.6.1.3); NOP-OR, nociceptin-orphanin receptor; PBS, phosphate-buffered saline; PM, plasma membrane, PMSF, phenylmethylsulfonyl fluoride; PNS, post-nuclear supernatant; PTX, pertussis toxin; RT-PCR, reverse transcription polymerase chain reaction; SLB; sample lyses buffer; w.w., wet weight; TBS, Tris-buffered saline; TCA, trichloroacetic acid.
